# 

**Published:** 1906-05

**Authors:** 


### CONTENTS.

ORIGINAL COMMUNICATIONS—

NEGRI BODIES IN RABIES. By James N. Brawner, M.D., Atlanta, Ga. .......... 73
CHRONIC CONSTIPATION. By L. Amster, M.D., Atlanta, Ga. .......... 80
TREATMENT OF CONSTIPATION. By Theodore Toepel, M.D., Atlanta, Ga. .......... 90

CORRESPONDENCE—

THE PENN MUTUAL LIFE INSURANCE COMPANY, PHILADELPHIA .......... 103

EDITORIAL NOTES AND COMMENTS—

AN UGLY FIGHT .......... 108

NEWS AND NOTES .......... 125

CONTENTS CONTINUED .......... X

